# Reduced cytotoxicity by mutation of lysine 590 of *Pseudomonas* exotoxin can be restored in an optimized, lysine-free immunotoxin

**DOI:** 10.1093/immadv/ltac007

**Published:** 2022-02-21

**Authors:** A Ammon, L Mellenthin, C Emmerich, E Naschberger, M Stürzl, A Mackensen, F Müller

**Affiliations:** 1 Department of Internal Medicine 5, Haematology and Oncology, University Hospital of Erlangen, Friedrich-Alexander University of Erlangen-Nuremberg (FAU), 91054 Erlangen, Germany; 2 Division of Molecular and Experimental Surgery, Translational Research Center, Department of Surgery, University Hospital of Erlangen, Friedrich-Alexander University of Erlangen-Nuremberg (FAU), 91054 Erlangen, Germany

**Keywords:** immunotoxin, antibody conjugate, *Pseudomonas* exotoxin, protein engineering, lysine-free protein

## Abstract

Immunotoxins, which are fusion proteins of an antibody fragment and a fragment of a bacterial or a plant toxin, induce apoptosis in target cells by inhibition of protein synthesis. ADP-ribosylating toxins often have few lysine residues in their catalytic domain. As they are the target for ubiquitination, the low number of lysines possibly prevents ubiquitin-dependent degradation of the toxin in the cytosol. To reduce this potential degradation, we aimed to generate a lysine-free (noK), *Pseudomonas* exotoxin (PE)-based immunotoxin. The new generation 24 kDa PE, which lacks all but the furin-cleavage site of domain II, was mutated at lysine 590 (K590) and at K606 in a CD22-targeting immunotoxin and activity was determined against various B cell malignancies *in vitro* and *in vivo*. On average, K590 mutated to arginine (R) reduced cytotoxicity by 1.3-fold and K606R enhanced cytotoxicity by 1.3-fold compared to *wild type* (*wt*). Mutating K590 to histidine or deleting K590 did not prevent this loss in cytotoxicity. Neither stability nor internalization rate of K590R could explain reduced cytotoxicity. These results highlight the relevance of lysine 590 for PE intoxication. In line with *in vitro* results, the K606R mutant was more than 1.8-fold more active than the other variants *in vivo* suggesting that this single mutation may be beneficial when targeting CD22-positive malignancies. Finally, reduced cytotoxicity by K590R was compensated for by K606R and the resulting lysine-free variant achieved *wt*-like activity *in vitro* and *in vivo*. Thus, PE24-noK may represent a promising candidate for down-stream applications that would interfere with lysines.

## Graphical Abstract

**Figure:**
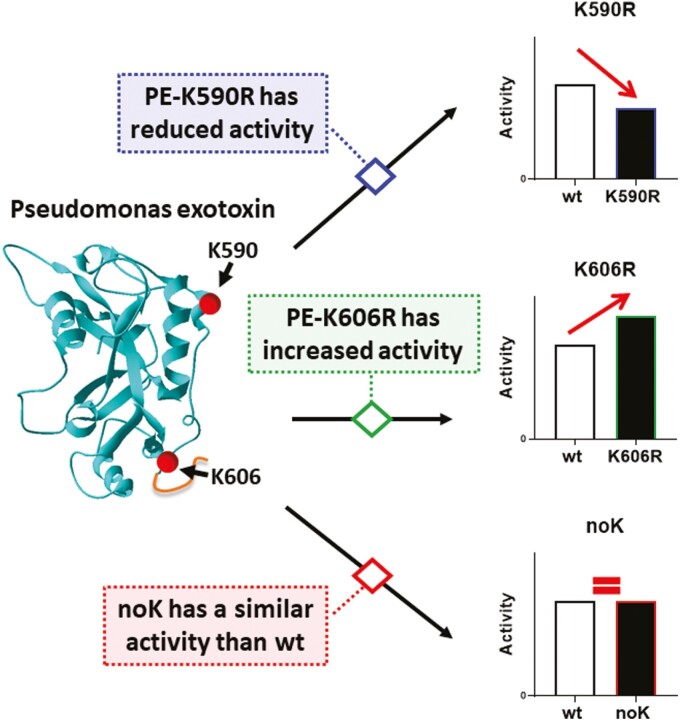

## Introduction

Monoclonal antibodies are widely used to target and modulate cells of interest. The cell of interest in oncology frequently is a cancer cell and antibodies are used to achieve cytotoxicity. For many targets though, the antibody itself is not active enough and is therefore linked with a cytotoxic agent. Immunotoxins are antibody fusion proteins genetically engineered to contain a bacterial or a plant toxin [1, 2]. The toxin component of immunotoxins targets translation either by cleaving a specific loop of the ribosomal RNA or by an enzymatic transfer of ADP-ribose to eukaryotic elongation factor 2 (eEF2) [3, 4]. Both events arrest protein synthesis – a unique mode of action that no other approved drug utilizes – and induce intrinsic apoptosis in the target cell. The immunotoxin-induced cell death produces a pro-immunogenic signal in preclinical models [5–7], which may explain clinically observed activation of anti-tumour immune responses by immunotoxins [8, 9].

Because it frequently achieves high cytotoxicity as a fusion partner, the 38 kDa, consisting of domain II and III of *Pseudomonas* exotoxin A (PE38), is studied widely [3]. One example is the CD22-targeting immunotoxin moxetumomab pasudotox, a fusion protein of an anti-CD22 antibody fragment and PE38. Moxetumomab is highly active against various B cell malignancies and is FDA-approved for the treatment of hairy cell leukaemia [10–12]. To improve cytotoxicity on average by two-fold and reduce immunogenicity, all but the furin-cleavage site of domain II can be deleted, resulting in a 24 kDa (PE24) toxin fragment [13, 14]. Thus, many novel PE-based immunotoxins are constructed using a PE24 derivate. Because the smaller protein has a shorter half-life, the variable fragment (Fv) of the antibody was exchanged with a larger antigen-binding fragment (Fab) which prolonged serum half-life and enhanced *in vivo* efficacy [14, 15].

**Figure 1.**
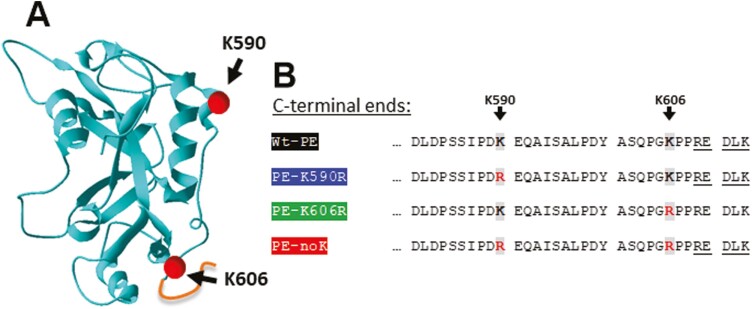
Structure and N-terminal sequence of Domain III of *Pseudomonas exotoxin* A. (A) Crystallographically resolved structure of Domain III of *Pseudomonas exotoxin* A derived from the protein data bank [50]. The pdb file was visualized using Swiss-PdbViewer [51]. K590 and K606 are highlighted as spheres in corresponding positions. The REDLK motif was added manually. (B) C-terminal sequence of indicated PE variants including *wild type* (*wt*), PE-K590R or PE-K606R where lysines (K in single amino acid code) at indicated positions were mutated to arginine (R). PE-noK contains both mutations K590R and K606R.

The route of ADP-ribosylating toxins from initial binding to their cell surface receptor to the cytosol is diverse [16, 17]. Several toxins, including PE, traverse to the endoplasmic reticulum (ER) [18]. A critical step in the intoxication is escaping ER so that PE can reach ribosomes in the cytosol. To escape the ER, PE uses an ER-associated degradation (ERAD) pathway, which includes binding to the translocon Sec61 (19, 20). When passing through the translocon, PE is believed to partially unfold and refold on the other side of the lipid bilayer membrane [16, 17]. Possibly because lysine-residues of unfolded proteins in the cytosol are rapidly ubiquitinylated and subsequently degraded in the proteasome, toxins that escape the ER through Sec61 have evolved to contain few lysines [16, 18]. In line with this hypothesis, remaining lysines may have an important function for passing the translocon or may be involved in the process of refolding once the toxin reaches the cytosol [17]. PE contains two lysine residues at positions 590 and 606 (Fig. 1A and B). Though few studies tested effects of simultaneous mutations of these two lysines in combination with additional PE-modifications such as a study describing PE38QQR, [21–23] effects on cytotoxicity of mutations of each lysine individually have not been studied before.

## Materials and methods

### Cell lines

Cell lines used: JeKo-1, Rec-1, KOPN8, and HAL-1. All cells were grown at 37°C, 5% CO_2_ in RPMI 1640 (Gibco™, Thermo Fisher Scientific), supplemented with 10% fetal bovine serum (FBS), 2mM L-glutamine, 100 U penicillin, and 100 mg streptomycin (Invitrogen, Carlsbad, CA, USA).

### Animal studies

All animal studies were approved by the Institutional Animal Care and Use Committee and adhered to the ARRIVE guidelines. Animals were handled according to institutional guidelines. Ten million JeKo-1 cells were injected intravenously (i.v.) at day 1 into the tail vein of NSG (NOD.Cg-Prkdc^scid^ Il2rg^tm1Wjl^/SzJ) mice. Mice were treated from day 15 with three doses of 1 mg/kg or with three doses of 0.4 mg/kg immunotoxin every other day (QOD). To analyse tumour infiltration, mice were euthanized at day 22 and bone marrow was isolated by flushing femurs. Cells were then mashed, washed with PBS (Gibco™), and stained with Zombie Aqua (BioLegend, Amsterdam, The Netherlands), Fc-block (anti-murine CD16/32, BioLegend), and stained with anti-CD22 FITC (BioLegend).

### Construction, expression and purification of immunotoxins

Plasmids for the heavy and the light chain of the *wild-type* (*wt*) immunotoxin were kindly provided by Dr Ira Pastan. For the PE mutant variants, codon-optimized gene blocks were ordered (IDT, Coralville, IA, USA) and cloned into the *wt* expression plasmid. Expression and purification of the immunotoxin were performed as described previously [49]. Briefly, immunotoxin heavy and light chains were expressed individually in BL21 *E. coli* (NEB, Ipswich, MA, USA), isolated as inclusion bodies, refolded for 32 h at pH 10.0 (11), dialyzed for two to three days, and then purified with a three-step chromatography protocol including two distinct ion exchange columns (Q Sepharose Fast Flow, Cytiva and Capto HiRes Q, Cytiva) followed by size exclusion column (Superdex 75 Increase GL, Cytiva) using an ÄKTApure system (Cytiva).

### Cell assays

For apoptosis assays, 100 000 cells/ml were plated into a 96-well format, treated with a serial dilution of the immunotoxin, and incubated for 72 h. Cells were then washed, stained with 7-AAD (BioLegend), and analysed by flow cytometry.

For time assays, 100 000 JeKo-1 cells/ml were plated into a 24-well format and incubated with 100 ng/ml immunotoxin for 1–4 h. After indicated time points, cells were washed twice and transferred to a new well with a fresh medium without immunotoxin. Cell viability was measured 72 h after initiation of the assay by flow cytometry as described above.

For stability assays, immunotoxins were left on ice or incubated for 5 min at 45°C. One hundred thousand JeKo-1 cells/ml were plated into a 96-well format and treated with a serial dilution of the heat-stressed immunotoxins and the respective control. Cell viability was measured after 72 h by flow cytometry.

For internalization and affinity measurements assays, immunotoxins were amine labelled with Alexa647 (Thermo Fisher Scientific, Waltham, MA, USA). Internalization was determined by incubation of 500 000 JeKo-1 cells/well for 0.5, 1, 2, or 3 h at 37°C with 1000 ng/ml Alexa647-immunotoxins. After the indicated time points, duplicate samples of each time point, and immunotoxin were washed and either left untreated or were surface stripped using glycine–pH 2.7 for 5 min. Then, samples were analyzed by flow cytometry. The internalization constant was generated using the affinity (KD) regression model of GraphPad Prism v9.0, whereas the algorithm incorporates estimated maximum binding signal (Bmax) and the time to reach half-maximum signal. For affinity measurement, 250 000 Jeko-1 cells were incubated with a serial dilution of immunotoxin in 0.1% sodium azide, 5% FBS, PBS on ice for 1 h. After incubation, cells were washed with PBS and analysed by flow cytometry.

### Statistics

All graphs and corresponding statistics were done using GraphPad Prism v9.0 and indicated statistical tests.

## Results

### Mutation of K606R increased cell killing

**Figure 2.**
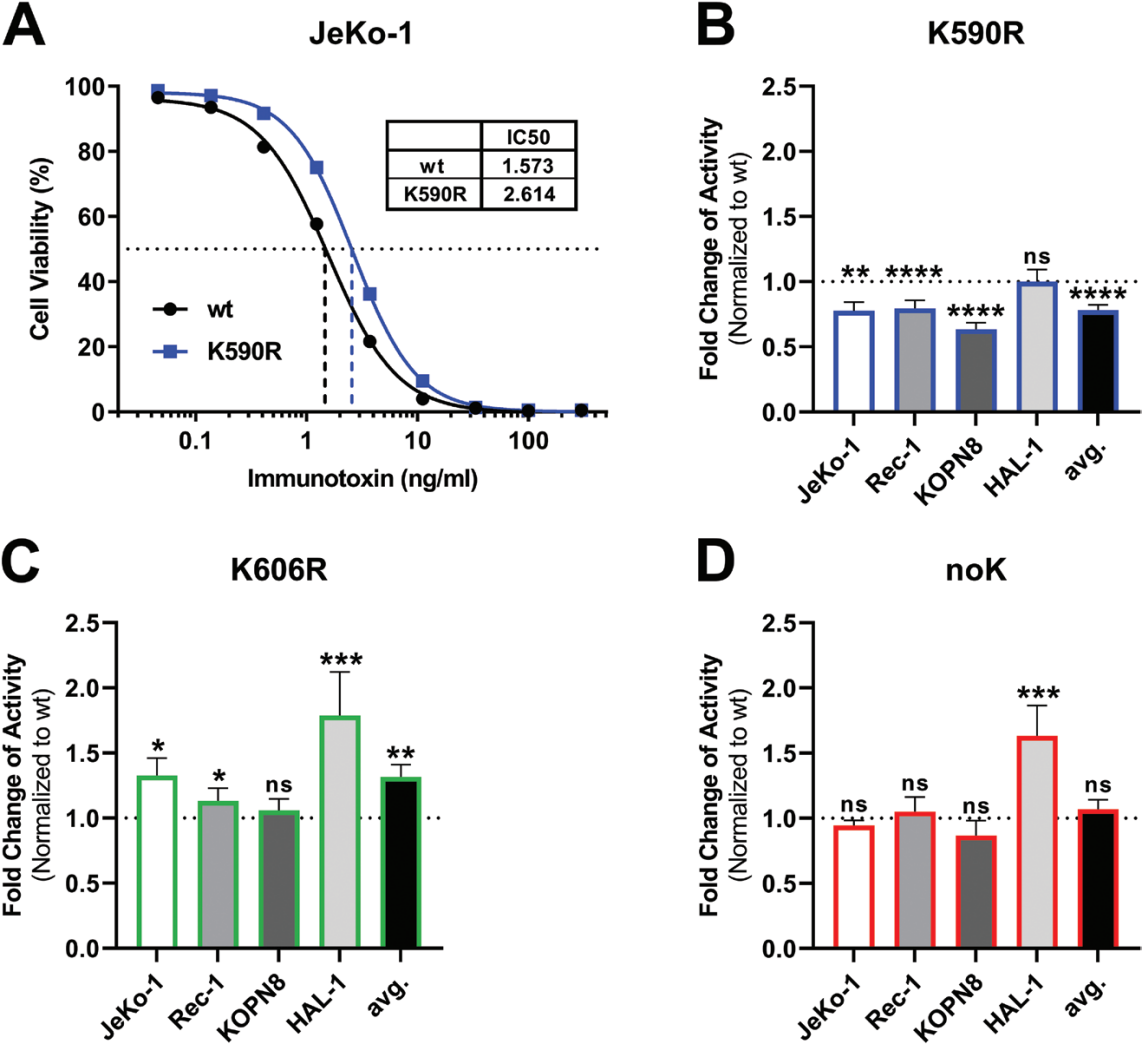
K606R increased immunotoxin activity. Various B cell malignancy cell lines were exposed to serial dilutions of indicated immunotoxin variants for 72 h and cell viability was analysed by flow cytometry. (A) *In vitro*-apoptosis assay comparing *wt* and K590R immunotoxin on JeKo-1 with correlating IC50s. Activity was defined as the reverse IC50. One representative experiment of *wt* and K590R is shown. (B–D) Fold change of activity of indicated variants compared to *wild type* immunotoxin against four B cell malignancy cell lines (K590R blue, K606R green, noK red). In addition, the mean of all fold-changes of all cell lines is indicated as ‘avg’. (ns: not significant, *P < 0.05, **P < 0.01, ***P < 0.001, ****P < 0.0001, unpaired *t*-test).

To analyse the role of K590 and of K606 for immunotoxin activity, we mutated them individually and simultaneously. Correlating immunotoxin variants are referred to as K590R, K606R, or noK (Fig. 1B). Cytotoxicity of these immunotoxin variants was analysed on different B cell malignancies including two mantle cell lymphoma cell lines (JeKo-1, Rec-1) and two B cell acute lymphoblastic leukaemia cell lines (KOPN8, HAL-1). Fig. 2A shows dose-dependent cell killing curves of the *wild type* (*wt*) and of the K590R immunotoxin against JeKo-1 cells. The inverse of half-maximal inhibitory concentration (IC50) was defined as activity and was normalized to the activity of *wt* immunotoxin. K590R was significantly less active than *wt* against JeKo-1, Rec-1, KOPN8, but not against HAL-1 (Fig. 2B). The average (avg.) activity of K590R overall cell lines was 78% compared with *wt* activity (*P* < 0.0001). The activity of K606R, on the other hand, was significantly higher against JeKo-1, Rec-1, and HAL-1 but not against KOPN8. Across all cell lines, K606R was more active on average by 1.3-fold than *wt* (Fig. 2C). The noK variant was similarly active against Jeko-1, Rec-1, and KOPN8 and was more active against HAL-1. On average across all cell lines, the IC50 of noK was not significantly different from *wt* (Fig. 2D).

### Lysine at 590 is needed for optimal immunotoxin activity

**Figure 3.**
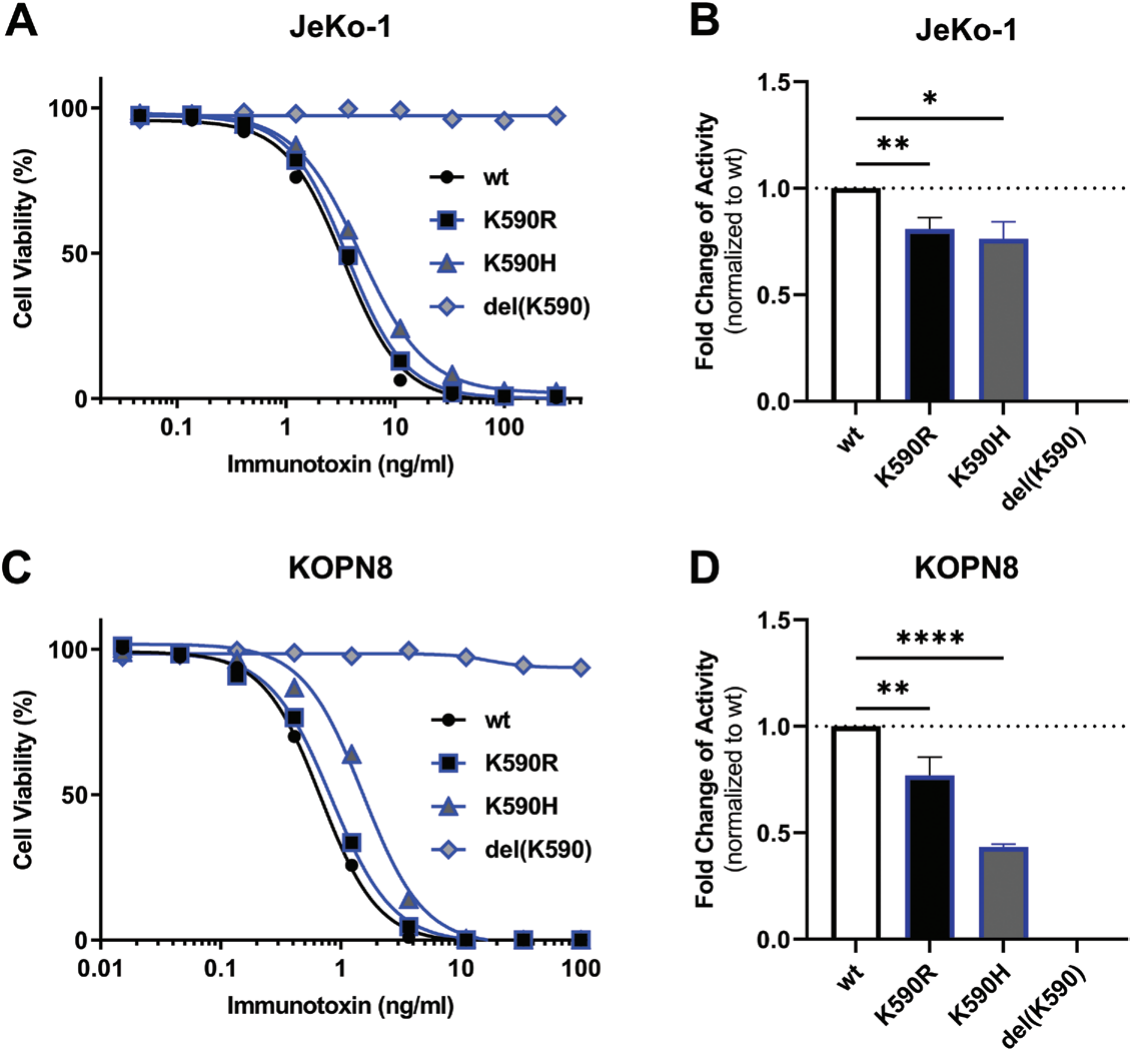
Alternate mutations other than arginine or a deletion of K590 further reduced cytotoxicity. Cells were exposed to indicated immunotoxin for 72 h and cell viability was analysed by flow cytometry. Shown is one representative viability graph for indicated immunotoxins against JeKo-1 (A) or KOPN-8 (C) and mean fold-change of activity of the indicated immunotoxin variants against JeKo-1 (B) or KOPN-8 (D). Bars represent the mean fold-change, error shown as SEM. Significance of difference from *wt* was determined by one-way ANOVA as ns: not significant, *P < 0.05, **P < 0.01, ***P < 0.001, ****P < 0.0001.

To investigate whether an alternate mutation of lysine 590 could prevent reduction of cytotoxicity induced by mutation to arginine, we mutated either lysine to histidine (K590H) or introduced a deletion of K590 (del(K590)). Del(K590) rendered the immunotoxin inactive against JeKo-1 (Fig. 3A and B). The mean activity of K590H compared to *wt* was, similar to that of K590R, reduced by 1.3-fold (*P* = 0.0119) against JeKo-1 cells. As for JeKo-1, del(K590) was inactive against KOPN8, while K590H was less active than K590R (*P* = 0.0087) and lost on average 2.3-fold activity compared to *wt* (*P* = <0.0001) (Fig. 3C and D).

### Shorter exposure time is needed for K606R to induce cell death

**Figure 4.**
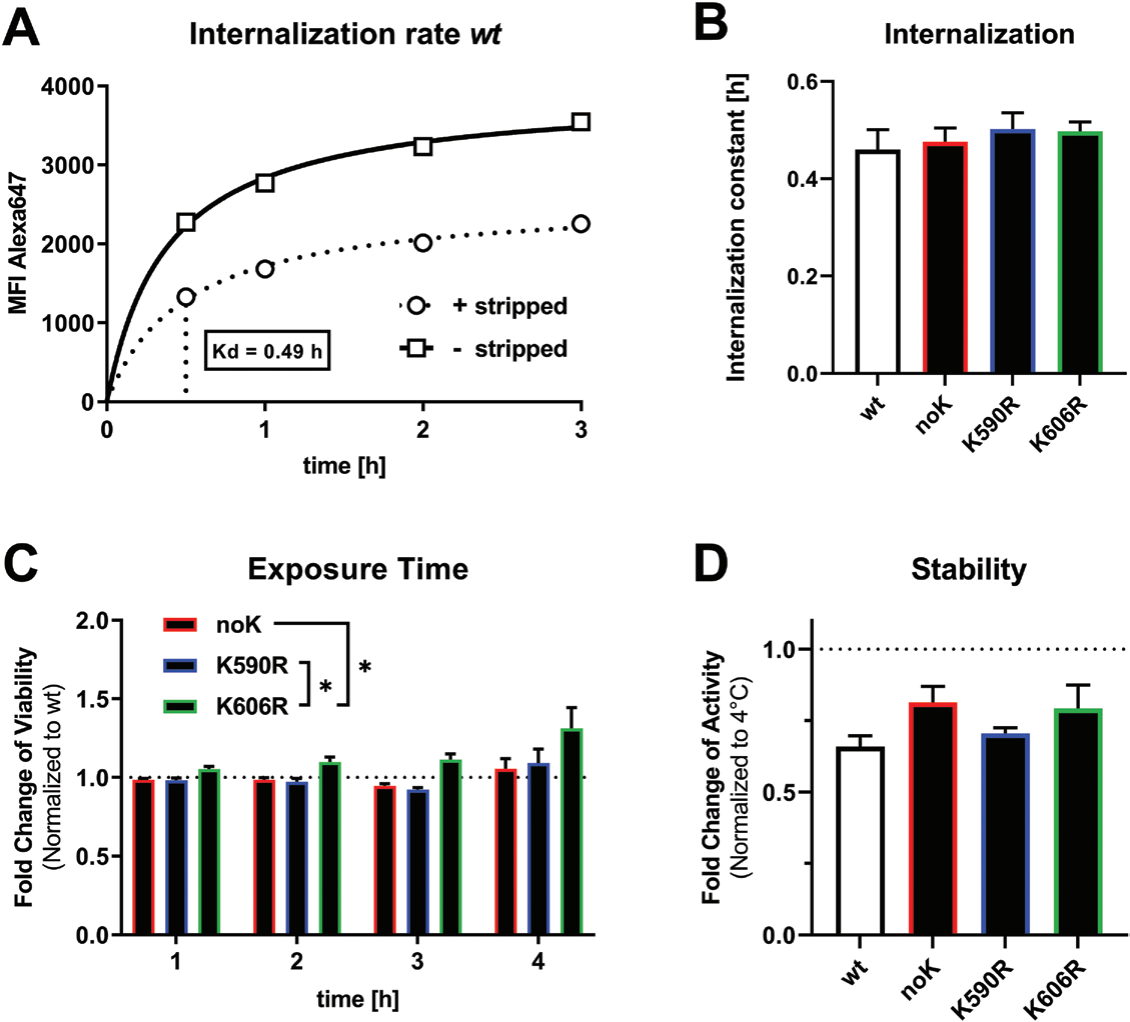
Shorter exposure to K606R results in higher cytotoxicity despite similar internalization rate and protein stability. (A) Rate of internalization on JeKo-l1 cells after incubation with 1000 ng/ml Alexa647-immunotoxin for 0.5, 1, 2, 3 h at 37°C. After indicated times, half the samples were surface stripped (representing only internalized immunotoxin) while the remainder were not (representing internalized and surface-bound immunotoxin), and cells were analysed by flow cytometry. Mean fluorescence intensity (MFI) is shown after background subtraction. (B) Mean internalization constant over time as an average of three independent experiments, error as SEM. Statistics by paired *t*-test determined differences were not statistically significant (ns). (C) JeKo-1 cells were incubated with 100 ng/ml of indicated immunotoxins for 1, 2, 3, or 4 h, washed and replated. After a total of 72 h from the initiation of the experiment, all cells were analysed by flow cytometry. Shown is relative viability normalized to *wt*. Bars represent mean fold-changes over three experiments, error shown as SEM. Significance was determined by one-way ANOVA, as ns: no significance, *P < 0.05. (D) Protein stability represented by cytotoxicity after exposure to 45°C for 5 min for each of the immunotoxin variants relative to the respective variant kept at 4°C. Cytotoxicity was determined against JeKo-1 cells and viability was determined by flow cytometry. Shown are means of biological duplicates from three individual experiments. Statistics were determined by unpaired *t*-tests and *P*-values indicated as ns: no significance, * < 0.05.

An explanation for differences in cytotoxicity could be a change in the internalization rate. To measure internalization, JeKo-1 cells were exposed for 0.5, 1, 2, or 3 h to the four immunotoxin variants conjugated with Alexa647. Total Alexa647-signal at each time point was compared to the Alexa647-signal of cells that were surface stripped, the latter signal correlating with the internalized immunotoxin. Fig. 4A shows a representative graph of Alexa647-signal over time with and without surface stripping. Using GraphPad Prism’s regression algorithm that usually determines the concentration for half-maximal binding concentration (or affinity (KD)), we determined an internalization constant that defined the time, which was needed to reach half-maximal internalization. The average internalization constant of the three immunotoxin variants was not significantly different from *wt* PE (Fig. 4B). In contrast, the internalization rate of the del(K590) mutant was more than 20-fold lower than that of *wt* (Supplementary Fig.1A). We, thus, tested for the quality of antibody refolding by determining the affinity of the del(K590) mutant (Supplementary Fig. 1B). Compared to *wt*, the affinity of del(K590) was not reduced. Thus, changes in the toxin domain but not of the antibody fragment is likely responsible for the inactivity of the del(K590) mutant.

It has previously been reported that the length of exposure time to immunotoxin correlates with cytotoxicity and improved trafficking to the cytosol [11]. We, therefore, incubated JeKo-1 cells for various time points with the distinct immunotoxin variants, washed and replated cells in a fresh medium, and analysed cell viability 72 h after initiation of the assay. Compared with *wt* immunotoxin at the individual exposure times, K606R tended to being more active than *wt*  (*P* = 0.063) and was significantly more active than K590R  (*P* = 0.046) and noK (*P* = 0.048) the longer the cells were exposed (Fig. 4C). Because of similar internalization rates, these data suggests that the K606R immunotoxin may reach eEF2 in the cytosol more efficiently.

The difference in activity could also be due to differences in protein stability. Therefore, we analysed cytotoxicity of the different immunotoxin variants after exposure to heat. Compared with the respective control immunotoxin variant kept at 4°C, all immunotoxins showed a similar loss in activity when heated to 45°C for 5 min. Thus, the respective mutations do not significantly alter stability and stability cannot explain the differences in activity.

### K606R shows a significantly increased efficacy in a JeKo-1 xenograft model

**Figure 5.**
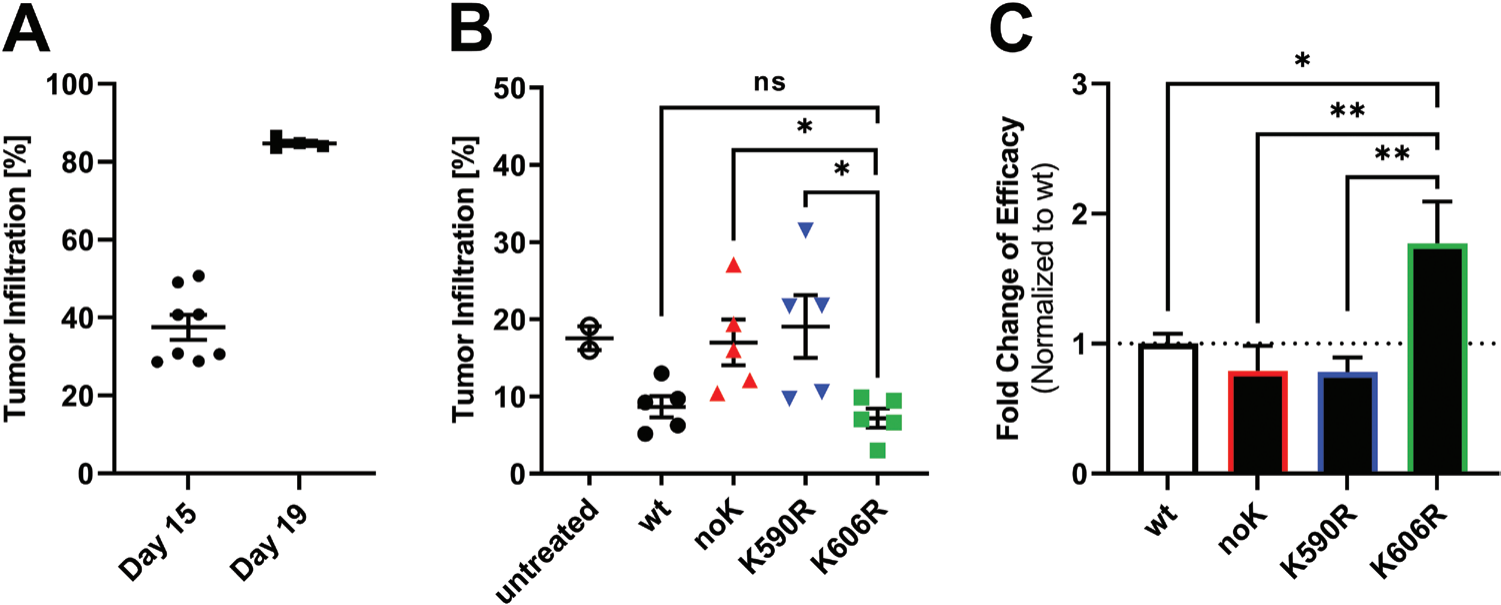
K606R is more active in a JeKo-1 xenograft model. (A) Growth kinetics after intravenous injection of 10 million JeKo-1 cells into NSG mice. Day 15 (d15) reflects average infiltration rate at treatment start and day 19 reflects infiltration rate at the last day of treatment. (B) Shown is one representative *in vivo* experiment of resulting bone marrow infiltration rates of JeKo-1 bearing mice after treatment with indicated immunotoxin variants. JeKo-1 cells were intravenously injected on day 1 and mice were treated from day 15 with three doses of 0.4 mg/kg of the indicated immunotoxin every other day. Three days after the last dose, bone marrow was isolated and analysed for lymphoma infiltration rate by flow cytometry (ns: no significance, *P < 0.025, unpaired *t*-test). (C) Mean fold-changes of the efficacy of four independent *in vivo* experiments in the JeKo-1 xenograft model were normalized to *wild-type* control. Bars indicate mean fold-change of efficacy, the error shown as SEM (*P < 0.05, ** < 0.01, one-way ANOVA).

Finally, to validate relevance of the lysine-mutants *in vivo*, we tested the immunotoxin variants in a systemic JeKo-1 xenograft model. In this model, three bolus doses of *wt* immunotoxin every other day previously achieved stabilization of tumour burden [10]. Fig. 5A shows tumour growth kinetics after intravenous (i.v.) injection of 10 million JeKo-1 cells on day 1. Untreated mice at day 15, defined as start of treatment, showed an average bone marrow infiltration rate of 38% which rose to 84% on day 19, which is the last day of treatment of three doses every other day starting at day 15. Having established tumour growth of the JeKo-1 model, we were interested in drug efficacy as defined by change of infiltration rate compared to treatment start on day 15. Untreated mice on day 15 showed an average JeKo-1 infiltration rate of 17.5% (Fig. 5B). Mice that were treated with three bolus doses of *wt* immunotoxin QOD from day 15 showed an average JeKo-1 bone marrow infiltration rate of 8.5% on day 21. Correlating with cytotoxicity *in vitro*, K590R was less active than *wt* and bone marrow infiltration rate was on average 19% on day 21 (*P* = 0.063). K606R was more active than *wt* and reduced bone marrow infiltration rate to 7% (Fig. 5B,  *P* = 0.36). To combine four individual *in vivo* experiments, the activity of *wt* was set to 1 and fold-change of efficacy was defined as inverse infiltration normalized to the *wt* (Fig. 5C). Compared to *wt*, the efficacy of noK was at 79%, of K590R at 78%, and of K606R at 177%, respectively. Hence, K606R was more active than *wt* (*P* = 0.0347), K590R (*P* = 0.0038), and noK (*P* = 0.0093) *in vivo* and was found to be the most active variant overall, both *in vitro* and *in vivo*.

## Discussion

*Pseudomonas* exotoxin A contains only two lysine residues in the catalytic domain and one lysine residue in its C-terminal KDEL-like motif REDLK. With the aim of generating a lysine-free immunotoxin, we studied the effects of K590 and K606 mutants on immunotoxin activity. K606R significantly improved cytotoxicity *in vitro* and *in vivo*, while K590R, on the other hand, reduced activity in three out of four tested cell lines. The loss of activity by K590R could neither be prevented by a mutation to histidine nor by a deletion of K590, whereas a simultaneous mutation of both lysines neutralized the effects of each individual K mutant on cytotoxicity resulting in the *wild type*-like activity of the noK variant.

### Relevance of lysines in PE

Several steps of the intoxication route of PE are well established. In line with commonly accepted key steps of intoxication, siRNA knock-down screens using full *Pseudomonas* exotoxin A or PE-based immunotoxins against distinct surface receptors consistently identify expected proteins as relevant for PE cytotoxicity [17, 24]. Among others, these proteins include KDEL-receptors that are highjacked by PE for retrograde trafficking to the ER and proteins needed to generate diphthamide, the target residue for ADP-ribosylation on eukaryotic elongation factor 2 (eEF2) [17, 24–26]. The relevance of other proteins involved in vesicular trafficking including SNAREs or rab proteins has shown higher variability with regard to PE cytotoxicity [17, 24]. This variability is likely explained by the redundant function of vesicle transport proteins in eukaryotic cells [27]. In addition to biologic heterogeneity, different types of surface receptors take distinct intracellular routes after internalization. We used a CD22-targeting immunotoxin. CD22 is internalized to the endocytic recycling compartment which allows highly efficient shuttling from the surface to the endosome [28, 29]. However, intracellular routes may be distinct for other immunotoxins targeting e.g. G-protein coupled receptors, type I/II membrane receptors including cytokine receptors, or GPI-linked proteins  [30–36]. Independent of the surface receptor, data suggest that all PE-based immunotoxins reach the ER and indicate that escaping ER is a critical step in PE intoxication.

Ribosome targeting toxins that reach the ER frequently highjack the ER-associated degradation (ERAD) pathway. Several lines of evidence suggest that PE uses the translocon (Sec61) to escape the ER which is different from other toxins including ricin that highjack DERLINs [17, 19, 37]. Similar to nascent proteins during translation, a freely accessible, C-terminal signal peptide with a short α-helix is believed to be involved in the initial binding step of PE to Sec61 [38]. K590 is located in the surface accessible C-terminal α-helix of PE and may be part of the initial binding motive of PE to Sec61 [19, 20, 39]. In line with a needed recognition motive, the del(K590) variant is inactive which may be explained by a disrupted α-helical structure [40]. That K590 is relevant for transport and not for the enzymatic function is further supported by data showing no change of ADP-ribosylation rate, the enzymatically catalysed reaction of PE, when K590 of PE38 is mutated to cysteine or to alanine [41, 42].

To pass the translocon and reach the cytosol, PE must, at least in part, unfold and then refold again once in the cytosol [17, 39]. For cytosolic refolding, the chaperon T-complex protein-1 ring complex (TRiC) is likely needed to regain PE activity [17, 43]. Assuming that lysine residues of unfolded PE would be rapidly ubiquitinated and then degraded by the proteasome, these lysines would counteract cytotoxicity [44]. In line with possible ubiquitination and degradation of PE after passing the translocon, knock-down of ubiquitin-supporting cullins mitigates PE toxicity [17, 44]. Following this hypothetical line of arguments, a reduced binding of K590R to the translocon and thus translocation to the cytosol may explain reduced cytotoxicity. On the other hand, mutated K590R and K606R may prevent ubiquitination and thus, degradation in the cytosol and therefore, enhance cytotoxicity, thus, together possibly explaining changes in cytotoxicity. Enhanced intracellular transport of PE and not altered enzymatic function are also supported by a shorter exposure time needed to kill the target cells as described for K606R in Fig. 3 [11, 30] .Additional work with genetically modified cells would be needed to prove these hypotheses.

### Comparison with other PE variants

We started searching for active PE-noK mutants with the goal to generate an immunotoxin for lysine-directed conjugation. Because the K of the *wild type* REDLK-motif is removed by carboxypeptidases before the toxin enters the cell, we did not modify the KDEL-motif [45]. Of note though, *wt*-REDLK of PE38 can be changed to KDEL or to RDEL without reducing cytotoxicity [46]. That mutation of K590 are possible as found here is in line with results from the Pastan group showing active immunotoxins that carry K590 mutations to cysteine, to glutamine, or to serine [42, 47, 48]. To achieve α-helical structure, two non-polar residues like alanine are frequently followed by a charged amino acid residue [40]. In line with the α-helical structure needed for the above postulated binding to Sec61, all reported substitute residues, including the histidine and arginine used here, are polar (charged side-chains). Similar to our data in a PE24 immunotoxin, K590 mutants of PE38 constructs published as individual mutations lost some activity [42, 47]. As found in this study, simultaneous mutations of both lysines at positions 606 and 590 result in a *wild type*-like activity also in a PE38 immunotoxin, thus allowing for a lysine-free PE with no reduction of cytotoxicity [21, 22]. In addition to its effects on cytotoxicity, K590 has been described as part of a B cell epitope [47]. Whether the change of lysine to arginine reduces immunogenicity as does a mutation of lysine 590 to alanine is not known.

In summary, our data in combination with the published literature suggest that the lysine at position 590 is relevant to achieve maximal cytotoxicity, whereas mutation of K606 enhances cytotoxicity of immunotoxins *in vitro* and *in vivo*. If needed for down-stream applications, a lysine-free mutant of PE24 can be used without significant loss compared to *wild type* PE.

## Supplementary material

Supplementary data are available at *Immunotherapy Advances* online.

**Supplementary Figure 1.** Internalization rate but not affinity of del(K590) is lower than of *wild type*. (A) Rate of internalization of indicated immunotoxins in JeKo-1 cells after incubation with 1000 ng/ml Alexa647-immunotoxin for 0.5, 1, 2, 3 h at 37°C. After indicated times, one half of the cells were surface stripped while the remainder were not. Then, cells were analysed by flow cytometry. The assay was performed in technical duplicates. (B) Affinity of *wild type* and del(K590) after 1 h incubation with a serial dilution of Alexa647-immuntoxin on JeKo-1 cells in 0.1% sodium azide, 5% FBS, PBS. The assay was performed in technical triplicates.

ltac007_suppl_Supplementary_Figure_S1

## Data Availability

The data underlying this article are available in the article and in its online supplementary material.

## Abbreviations

eEF2: eukaryotic elongation factor 2
Fab: antigen-binding fragment
Fv: variable fragment
noK: lysine-free
PE: *Pseudomonas* exotoxin
QOD: every other day
