# Supplementary figures and images for: Reduced cytotoxicity by mutation of lysine 590 of Pseudomonas exotoxin can be restored in an optimized, lysine-free immunotoxin
Source: Immunother Adv. 2022 Feb 21;2(1):ltac007. doi: 10.1093/immadv/ltac007 (PMC9327129; doi:10.1093/immadv/ltac007)

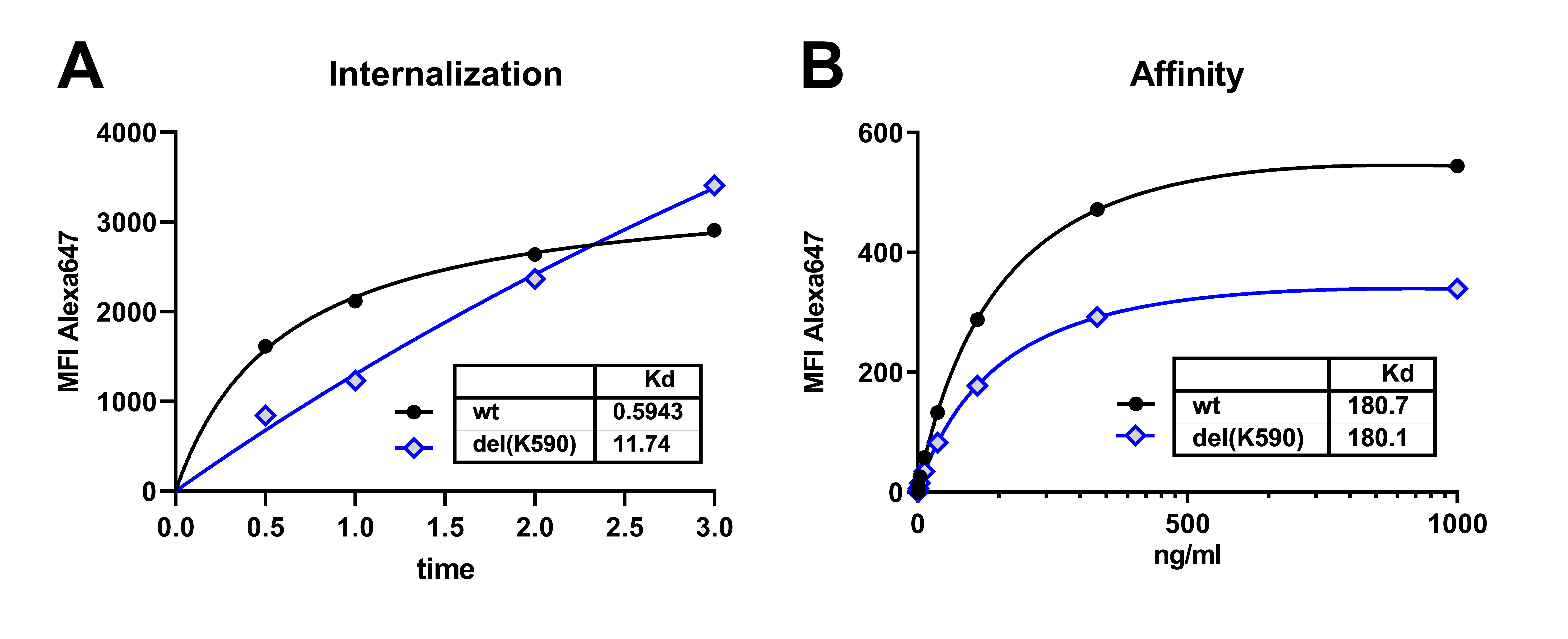

Supplement: ltac007_suppl_Supplementary_Figure_S1 [file ltac007_suppl_supplementary_figure_s1.jpeg]
